# The Relationship between Total Bilirubin Levels and Total Mortality in Older Adults: The United States National Health and Nutrition Examination Survey (NHANES) 1999-2004

**DOI:** 10.1371/journal.pone.0094479

**Published:** 2014-04-11

**Authors:** Kwok-Leung Ong, Matthew A. Allison, Bernard M. Y. Cheung, Ben J. Wu, Philip J. Barter, Kerry-Anne Rye

**Affiliations:** 1 Centre for Vascular Research, University of New South Wales, Sydney, New South Wales, Australia; 2 Faculty of Medicine, University of Sydney, Sydney, New South Wales, Australia; 3 Department of Family and Preventive Medicine, University of California San Diego, La Jolla, California, United States of America; 4 Department of Medicine, University of Hong Kong, Hong Kong, China; University of New South Wales, Australia

## Abstract

**Objective:**

Due to its anti-oxidant and anti-inflammatory properties, bilirubin has been associated with reduced cardiovascular risk. A recent study demonstrated an L-shaped association of pre-treatment total bilirubin levels with total mortality in a statin-treated cohort. We therefore investigated the association of total bilirubin levels with total mortality in a nationally representative sample of older adults from the general population.

**Methods:**

A total of 4,303 participants aged ≥60 years from the United States National Health and Nutrition Examination Survey 1999–2004 with mortality data followed up through December 31, 2006 were included in this analysis, with a mean follow-up period of 4.5 years.

**Results:**

Participants with total bilirubin levels of 0.1–0.4 mg/dl had the highest mortality rate (19.8%). Compared with participants with total bilirubin levels of 0.5–0.7 mg/dl and in a multivariable regression model, a lower total bilirubin level of 0.1–0.4 mg/dl was associated with higher risk of total mortality (hazard ratios, 1.36; 95% confidence interval, 1.07–1.72; P = 0.012), while higher levels (≥0.8 mg/dl) also tended to be associated with higher risk of total mortality, but this did not reach statistical significance (hazard ratios, 1.24; 95% confidence interval, 0.98–1.56; P = 0.072).

**Conclusion:**

In this nationally representative sample of older adults, the association of total bilirubin levels with total mortality was the highest among those with a level between 0.1 and 0.4 mg/dl. Further studies are needed to investigate whether higher total bilirubin levels could be associated with a higher mortality risk, compared to a level of 0.5–0.7 mg/dl.

## Introduction

Bilirubin is produced by the action of the enzyme biliverdin reductase on biliverdin during the degradation of heme [1]. Heme oxygenase (HO), the rate-limiting enzyme in the degradation of heme to biliverdin, carbon monoxide and ferritin, has been suggested as a potential therapeutic target in vascular diseases [1]–[3]. For example, HO-1, the inducible isoform of HO, plays a role in vascular repair by increasing circulating endothelial progenitor cells [4]. Induction of HO-1 expression can also improve vascular dysfunction in animal models of atherosclerosis, thrombosis, myocardial infarction, and hypertension [3].

Notably, bilirubin has anti-oxidant and anti-inflammatory effects, and can protect serum lipids from oxidation [1]. In this regard, previous studies have suggested bilirubin to be a protective biomarker for cardiovascular risk [1]. For example, lower total bilirubin levels are associated with higher risk of the metabolic syndrome [5], coronary artery disease (CAD) [6], peripheral arterial disease [7], stroke [8], and other cardiovascular diseases (CVDs) [1]. Higher total bilirubin levels (within the normal range) are also associated with a lower risk of cancer [9] and decreased cancer mortality [10]. Among patients from primary care practices, higher total bilirubin levels are also associated with a lower risk of respiratory diseases and total mortality [11].

In a recent prospective study of a large, statin-treated cohort, total bilirubin levels measured 3 months before statin treatment were found to be associated with any CVD event, coronary heart disease, myocardial infarction, and all-cause mortality in a non-linear and L-shaped relationship [12]. However, it is not known whether such non-linear L-shaped associations are apparent in a nationally representative sample of the general population. Moreover, there is a lack of studies on this association in older adults, who are at a high mortality risk due to aging. We therefore examined the association of total bilirubin levels with total mortality in older adults using data from the US National Health and Nutrition Examination Survey (NHANES). We also investigated whether such associations differed by demographic and lifestyle factors that were associated with total bilirubin levels.

## Subjects and Methods

### Study subjects

NHANES was conducted by the National Center for Health Statistics of the Centers for Disease Control and Prevention as a continuous cross-sectional survey of the health and nutritional status of the civilian, non-institutionalized United States population [13]. In 1999, the survey became a continuous program and examined a nationally representative sample of about 5,000 people each year. Data were released for every two-year cycle. The detailed measurement procedures and protocols have been described elsewhere [13]. All participants gave informed consent and the study received approval from the Centers for Disease Control and Prevention Institutional Review Board.

In NHANES 1999–2004 there were 4,984 participants aged ≥60 years who were both interviewed and examined in the mobile examination center. As total bilirubin levels are often elevated in liver diseases, 678 subjects with abnormal liver function (defined as a serum aspartate aminotransferase or alanine aminotransferase >100 U/l, a serum γ-glutamyltransferase >100 U/l, or total bilirubin level >3 mg/dl) [14], [15], or a self-reported history of liver diseases were excluded from the analysis to avoid the confounding effect of liver diseases, including those with subclinical and undiagnosed diseases. Among the remaining 4,306 participants, 4,303 participants had mortality data followed up through December 31, 2006 with a mean follow-up period of 4.5 years (range 0 to 92 months).

### Variables of interest

Mortality information was based on the results from a probabilistic match between NHANES and National Death Index death certificate records [16]. The causes of death were grouped into a standardized list of 113 causes based on the International Classification of Diseases, Tenth Revision (ICD-10). This allowed the number of deaths from CVDs (ICD-10 codes I00-I78 and I80-I99), cancer (ICD-10 codes C00–D48), and liver disease (ICD-10 codes B15-B19, K70, and K73-K74) to be estimated. Briefly, total bilirubin levels were measured as part of the routine liver function test using an Hitachi Model 704 multichannel analyzer (Boehringer Mannheim Diagnostics, Indianapolis, IN) in 1999–2000, a Beckman Synchron LX20 system in 2003–2007 and a Beckman Synchron DxC800 system (Beckman Coulter Inc, Fullerton, CA) in 2008–2010. In 2001–2002, these levels were measured using either an Hitachi Model 704 multichannel analyzer or a Beckman Synchron LX20 system. The reported values have been adjusted by regression equations to allow comparison across the two methods [13].

### Other covariates

Information on race/ethnicity, education, smoking, alcohol consumption, medications for hypertension and diabetes, history of CVD (defined as heart attack, congestive heart failure, coronary heart disease, angina, or stroke), arthritis and cancer were obtained from self-reported questionnaires at baseline. Body mass index (BMI) was calculated as weight in kilograms divided by the square of height in meters. Ever smokers were defined as subjects who had smoked ≥100 cigarettes in their lives. Regular alcohol consumption was defined as consumption of any type of alcoholic beverage at least once a week in the past year. Hypertension was defined as blood pressure ≥140/90 mm Hg or taking anti-hypertensive medications. Diabetes was defined as a history of physician diagnosis, non-fasting glucose ≥200 mg/dl, glycated hemoglobin ≥6.5%, or being treated with insulin or oral glucose-lowering medications. Data on the usage of different classes of lipid-lowering medication (including statins, fibrates, nicotinic acid, and other classes) and anti-hypertensive medication (including angiotensin-converting enzyme inhibitors [ACEI]/angiotensin receptor blockers [ARB], diuretics, β-blockers, calcium channel blockers [CCB], and others) in the past month were obtained from questionnaires of prescription medications as described [15], [17], [18]. The estimated glomerular filtration rate (eGFR) was calculated using the modified prediction equation from the Modification of Diet in Renal Disease study [19]. Albuminuria was defined as a urinary albumin-to-creatinine ratio ≥30 µg/mg [20]. Total white blood cell counts and hemoglobin levels were determined using the Beckman Coulter MAXM instrument (Beckman Coulter Inc, Fullerton, CA). Details on the laboratory analytical methods of other clinical biochemical parameters have been described previously [13], [15], [17], [18].

### Statistical analysis

Data analysis was performed using the complex sampling function of SPSS version 21.0 (SPSS Inc, Chicago, IL). Data are expressed as mean, percent (standard error), or geometric mean (95% confidence interval [CI]). All variables had less than 5% missing data (Supplementary Table S1). To obtain estimates representative of the United States Census civilian non-institutionalized population, examination sampling weights were used in all analyses to adjust for non-response bias and the oversampling of blacks and Mexican Americans [13]. Sampling errors were estimated using the primary sampling units and strata provided in the dataset.

Cox proportional hazards regression was used to estimate the hazard ratios (HR) for total mortality. As most participants (n = 952) had a total bilirubin level of 0.6 mg/dl (i.e. 10 µmol/l), this level was used as the referent group for comparison in the initial analysis (multicategory model A). In the recent study of Horsfall et al [12], total bilirubin level was transformed into restricted cubic spines with 4 knots at 0.3 mg/dl (5 µmol/l), 0.5 mg/dl (9 µmol/l), 0.7 mg/dl (12 µmol/l), and 1.2 mg/dl (20 µmol/l) [12]. Therefore in a separate analysis (multicategory model B), we divided the subjects into 3 groups (0.1–0.4, 0.5–0.7 and ≥0.8 mg/dl as the number of subjects <0.3 and >1.2 are small (n = 38 and 172 respectively). To account for the variations that may be due to changes in measurement sites and methods, the survey year was included in the regression model as a categorical independent variable. Age, sex, race/ethnicity, body mass index, and other variables, that showed significant difference between participants who were alive and those who were decreased (i.e. those with P<0.05 in Table 1) or correlated with total bilirubin levels (i.e. those with P<0.05 in Supplementary Table S1) were used as covariates in the Cox regression analysis. No multi-collinearity issue was detected as assessed by the variance inflation factors (<3.0 for all covariates). The *P* values for interaction were estimated by including each multiplicative interaction term in the regression models in full sample after adjusting for the main effects of the covariates. To assess the potential non-linear U-shaped relationship between total bilirubin and total mortality, total bilirubin values were entered as both a linear and quadratic terms at the same time in the Cox regression model and after full adjustment [21]. A two-tailed P<0.05 was considered statistically significant.

**Table 1:** Clinical Characteristics in United States Older Adults by Mortality Status, 1999–2004.

Characteristics	Alive (n = 3603)	Deceased (n = 700)	P^c^
Demographic and lifestyle factors			
Age, y	70.3 (0.2)	76.2 (0.4)	<0.001
Women, %	58.3 (0.8)	48.1 (2.3)	<0.001
BMI, kg/m^2^	28.4 (0.1)	27.1 (0.3)	0.12
Race/ethnicity, %			0.007
Non-Hispanic White	81.8 (1.8)	82.5 (2.3)	
Non-Hispanic Black	7.4 (1.0)	9.7 (1.6)	
Mexican American	3.2 (0.8)	2.4 (0.6)	
Others	7.6 (1.2)	5.5 (1.6)	
Education, %			<0.001
<High school	28.4 (1.5)	38.8 (2.1)	
High school diploma	28.9 (1.1)	29.6 (2.1)	
>High school	42.7 (1.4)	31.7 (2.8)	
Smoking, %			<0.001
Never	48.2 (1.2)	39.4 (2.2)	
Former	41.3 (1.1)	43.0 (2.0)	
Current	10.5 (0.6)	17.5 (2.4)	
Regular alcohol consumption, %	26.5 (1.7)	19.3 (1.7)	0.005
Chronic conditions			
History of CVD, %	20.9 (1.0)	42.5 (2.1)	<0.001
Diabetes, %	17.7 (1.0)	20.5 (1.6)	0.034
Hypertension, %	65.5 (1.1)	72.4 (2.0)	0.39
Albuminuria,%	17.0 (0.8)	40.4 (2.3)	<0.001
Arthritis, %	49.2 (1.0)	52.9 (2.3)	0.28
Cancer, %	19.8 (0.7)	28.6 (1.9)	0.007
Lipid-lowering medication, %	27.1 (0.9)	22.0 (2.2)	0.50
Statin	25.1 (0.8)	19.0 (2.1)	0.19
Fibrate	1.7 (0.2)	2.7 (0.8)^d^	0.011
Nicotinic acid	0.4 (0.1)	0.8 (0.5)^d^	0.68
Others^a^	1.3 (0.3)	0.2 (0.2)^d^	0.15
Anti-hypertensive medication, %	54.8 (1.3)	66.7 (2.4)	0.002
ACEI/ARB	28.0 (1.3)	30.6 (1.8)	0.002
Diuretic	24.6 (1.0)	33.7 (2.4)	0.019
β-blocker	18.5 (0.8)	21.4 (2.2)	0.052
CCB	17.7 (0.7)	24.2 (1.7)	0.11
Others	9.9 (0.6)	16.9 (2.1)	0.002
Clinical biomarkers			
Total cholesterol, mg/dl	211.2 (0.8)	209.1 (2.1)	0.63
HDL cholesterol, mg/dl	54.4 (0.5)	53.5 (0.7)	0.96
Serum albumin, g/dl	4.24 (0.01)	4.16 (0.02)	<0.001
Blood urea nitrogen, mg/dl^b^	15.3 (15.0–15.6)	18.5 (17.8–19.2)	<0.001
eGFR, ml/min/1.73 m^2^	71.2 (0.5)	62.7 (1.0)	<0.001
C-reactive protein, mg/dl^b^	0.25 (0.24–0.26)	0.35 (0.32–0.38)	<0.001
Alkaline phosphatase, U/l^b^	70.8 (69.7–72.0)	76.3 (74.3–78.3)	0.002
Alanine aminotransferase, U/l^b^	20.3 (19.9–20.7)	17.8 (17.2–18.4)	<0.001
Aspartate aminotransferase, U/l^b^	22.9 (22.6–23.2)	22.6 (22.0–23.2)	0.51
γ-glutamyltransferase, U/l^b^	21.5 (20.9–22.1)	21.6 (20.7–22.6)	0.026
Uric acid, mg/dl	5.59 (0.03)	6.08 (0.09)	<0.001
White blood cell count, 10^3^cells/µl^b^	6.78 (6.69–6.87)	7.31 (7.08–7.55)	<0.001
Hemoglobin, g/dl	14.3 (0.1)	13.9 (0.1)	<0.001
Total bilirubin, mg/dl^b^	0.66 (0.64–0.67)	0.62 (0.60–0.64)	0.078

ACEI  =  angiotensin-converting enzyme inhibitor; ARB  =  angiotensin receptor blocker; BMI  =  body mass index; CCB  =  calcium channel blocker; CI  =  confidence interval; CVD  =  cardiovascular disease; eGFR  =  estimated glomerular filtration rate; HDL  =  high-density lipoprotein; SE  =  standard error.

Data are expressed as mean or percent (standard error), unless otherwise noted.

a Includes bile acid sequestrants, cholesterol adsorption inhibitors, and other types of lipid-lowering medications.

b Data are expressed as geometric mean (95% CI).

c Estimated from the Cox regression model after adjusted for age, sex, race/ethnicity, and survey period, where appropriate.

d Estimates are unreliable due to coefficient of variation > 0.3.

## Results

Among the 4,303 participants, there were 700 deaths identified with a weighted mortality rate of 14.2% over a mean follow-up period of 4.5 years. Cause of death was identified in 692 of 700 subjects, with 270 deaths from CVD, 169 deaths from cancer and 253 deaths from other causes (including 3 deaths from liver disease). Table 1 shows the baseline characteristics of the participants according to the mortality status. Compared to participants who were alive, those who were deceased were more likely to be older, male, non-Hispanic Black, less educated, current smokers, and not regular alcoholic drinkers, with higher prevalence of CVD, diabetes, albuminuria, and cancer. They were also more likely to take anti-hypertensive medications, especially ACEI/ARB and diuretics, and have higher blood urea nitrogen, C-reactive protein, alkaline phosphatase, γ-glutamyltransferase, uric acid and white blood cell count, and lower serum albumin, eGFR, alanine aminotransferase and hemoglobin levels.

Supplementary Table S1 shows the clinical characteristics when total bilirubin levels were divided into six categories with similar numbers of participants. Participants with lower total bilirubin levels were younger, female, non-Hispanic Black, current smokers, and not regular alcohol drinkers; they also had a higher prevalence of diabetes, with lower serum albumin, alanine aminotransferase, aspartate aminotransferase, uric acid and hemoglobin levels, but higher levels of high-density lipoprotein (HDL) cholesterol, C-reactive protein, alkaline phosphatase and total white blood cell counts. The prevalence of self-reported CVD also tended to decrease with higher total bilirubin, although the highest prevalence was found in participants with a total bilirubin level of ≥1.0 mg/dl. Participants with higher total bilirubin levels tended to take lipid-lowering medications such as statins, but the differences did not reach statistical significance. They were less likely to take CCB.

Table 2 shows the total mortality rates in participants according to six different categorical groups of total bilirubin levels. The highest mortality rate was found in participants with total bilirubin levels of 0.1–0.4 mg/dl, although there was no significant difference in baseline total bilirubin levels between participants who were alive and those who were deceased (P = 0.078, Table 1). In the multivariable Cox regression model, a total bilirubin level of 0.1–0.4 mg/dl was associated with a higher risk of total mortality, compared to a level of 0.6 mg/dl after adjusting for confounding factors (P = 0.014, multicategory model A, Table 3). Compared to a level of 0.6 mg/dl, a total bilirubin of ≥0.8 mg/dl also tended to be associated with a higher risk of total mortality, although the association did not reach statistical significance. Similar results were obtained in the Cox regression analysis when total bilirubin levels were divided into three groups, 0.1–0.4, 0.5–0.7 and ≥0.8 mg/dl (multicategory model B, Table 3).

**Table 2:** Percentages of Total Death in United States Older Adults, 1999–2004.

Total bilirubin, mg/dl	Number at risk^a^	Total death	
		Number^a^	% (SE)
0.1–0.4	605	129	19.8 (1.6)
0.5	655	130	15.6 (1.5)
0.6	952	137	12.6 (1.2)
0.7	669	97	12.8 (1.8)
0.8–0.9	832	125	13.4 (1.3)
≥1.0	590	82	12.3 (1.4)

SE  =  standard error.

a Unweighted numbers.

**Table 3:** Association of Total Bilirubin Levels With Total Mortality in United States Older Adults, 1999–2004.

Total bilirubin, mg/dl	Model 1^a^		Model 2^b^		Model 3^c^		Model 4^d^	
	HR (95% CI)	P	HR (95% CI)	P	HR (95% CI)	P	HR (95% CI)	P
Multicategory model A								
0.1–0.4	1.57 (1.14–2.16)	0.007	1.53 (1.06–2.20)	0.024	1.68 (1.21–2.32)	0.002	1.51 (1.09–2.09)	0.014
0.5	1.33 (0.98–1.79)	0.063	1.13 (0.81–1.59)	0.45	1.18 (0.83–1.68)	0.34	1.19 (0.85–1.68)	0.31
0.6	1.00 (referent)		1.00 (referent)		1.00 (referent)		1.00 (referent)	
0.7	1.03 (0.80–1.33)	0.81	1.05 (0.79–1.39)	0.73	1.06 (0.76–1.49)	0.73	1.18 (0.86–1.61)	0.30
0.8–0.9	1.09 (0.86–1.38)	0.46	1.16 (0.90–1.50)	0.25	1.18 (0.87–1.59)	0.27	1.35 (1.00–1.83)	0.051
≥1.0	0.98 (0.69–1.39)	0.90	1.20 (0.84–1.73)	0.31	1.18 (0.80–1.73)	0.40	1.38 (0.98–1.94)	0.061
Overall P		0.10		0.14		0.013		0.022
Multicategory model B								
0.1–0.4	1.39 (1.09–1.78)	0.010	1.44 (1.10–1.88)	0.009	1.55 (1.23–1.96)	<0.001	1.36 (1.07–1.72)	0.012
0.5–0.7	1.00 (referent)		1.00 (referent)		1.00 (referent)		1.00 (referent)	
≥0.8	0.96 (0.79–1.18)	0.70	1.12 (0.91–1.39)	0.28	1.11 (0.89–1.37)	0.36	1.24 (0.98–1.56)	0.072
Overall P		0.030		0.031		0.002		0.008

HR  =  hazard ratio; CI  =  confidence interval.

a Adjusted for survey period, age, sex, and race/ethnicity (n = 4,303).

b Further adjusted for body mass index, education, smoking, and regular alcohol consumption (n = 3,928).

c Further adjusted for history of cardiovascular disease, diabetes, albuminuria, cancer, fibrates, angiotensin-converting enzyme inhibitors/angiotensin receptor blockers, diuretics, and calcium channel blockers (n = 3,764).

d Further adjusted for high-density lipoprotein cholesterol, serum albumin, blood urea nitrogen, estimated glomerular filtration rate, C-reactive protein, alkaline phosphatase, alanine aminotransferase, aspartate aminotransferase, γ-glutamyltransferase, uric acid, white blood cell count, and hemoglobin (n = 3,758).

**Figure 1:**
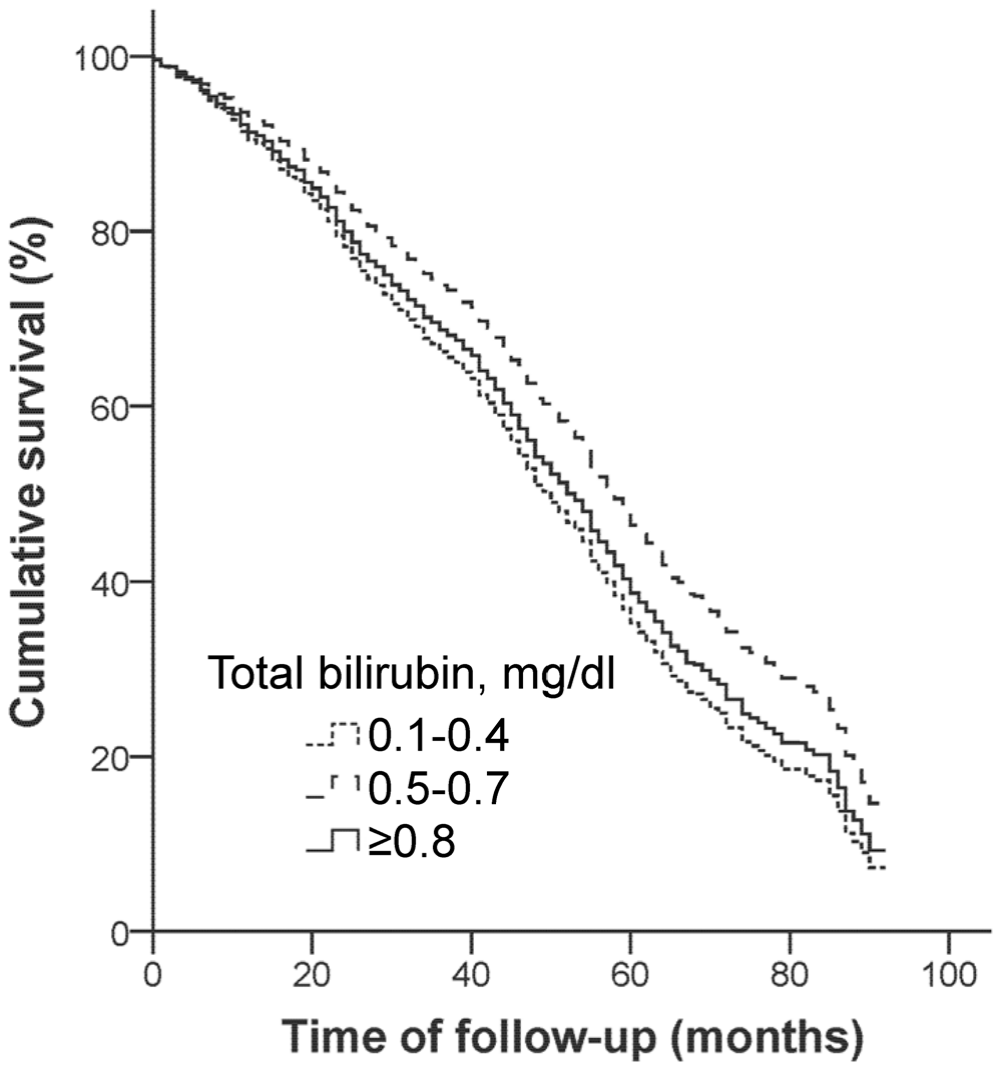
The cumulative survival curve for total mortality from the Cox regression model after full adjustment (model 4 of Table 3).

Figure 1 shows the adjusted cumulative survival curves. The survival curves of the total bilirubin levels of 0.1–0.4 mg/dl and ≥0.8 mg/dl diverged from that of a level of 0.5–0.7 mg/dl throughout the follow-up period without overlapping. In a test for the non-linear U-shaped relationship fitting a linear and quadratic terms of total bilirubin, a trend of U-shaped relationship was suggested by a marginal non-significant P value for the quadratic term (P = 0.066). In the analysis with cause-specific mortality (Supplementary Table S2), compared to a level of 0.5–0.7 mg/dl total bilirubin, a total bilirubin level of 0.1–0.4 mg/dl tended to be associated with higher cancer mortality (HR, 1.94; P = 0.016), whereas a total bilirubin level of ≥0.8 mg/dl tended to be associated more with mortality unrelated to CVD and cancer (HR, 1.88; P = 0.002).

A significant interaction between race/ethnicity and total bilirubin levels was found for total mortality (P for interaction = 0.008). In non-Hispanic Whites, both lower and higher levels of total bilirubin were significantly associated with a higher mortality risk (Table 4). There was also a significant interaction between smoking and total bilirubin levels for total mortality (P for interaction = 0.047). Among current smokers, a more linear association was found, in which lower total bilirubin levels were associated with higher mortality risk, and higher levels were associated with lower risk (Table 4). Among never smokers, higher levels of total bilirubin were more strongly associated with higher risk of total mortality than lower levels of total bilirubin. No significant interaction was found for age, gender and alcohol drinking (Supplementary Table S3). As shown in Supplementary Table S4, non-Hispanic Whites tended to have the highest prevalence of self-reported CVD and usage of lipid-lowering medications.

**Table 4:** Associations of Lower and Higher Total Bilirubin Levels With Total Mortality by Race/ethnicity and Smoking Status in United States Older Adults, 1999–2004.^a^

Subgroup	n	Mortality rate, % (SE)	0.1–0.4 mg/dl		≥0.8 mg/dl		Overall P
			HR (95% CI)	P	HR (95% CI)	P	
Race/ethnicity							
Non-Hispanic White	2511	14.3 (1.0)	1.52 (1.145–2.03)	0.006	1.38 (1.06–1.80)	0.018	<0.001
Non-Hispanic Black	655	17.8 (2.0)	1.13 (0.59–2.17)	0.71	1.17 (0.58–2.37)	0.66	0.86
Mexican American	879	10.8 (1.6)	1.11 (0.61–2.02)	0.74	0.46 (0.19–1.10)	0.081	0.21
Others	258	10.6 (1.8)	0.21 (0.02–1.82)	0.15	0.21 (0.02–2.85)	0.24	0.25
Smoking							
Never	2024	12.0 (0.9)	1.28 (0.80–2.05)	0.29	1.62 (1.08–2.43)	0.020	0.048
Former	1765	14.7 (0.9)	0.98 (0.61–1.57)	0.93	1.22 (0.84–1.76)	0.29	0.52
Current	505	21.7 (2.8)	2.55 (1.39–4.67)	0.003	0.59 (0.25–1.41)	0.23	0.002

CI  =  confidence interval; HR  =  hazard ratio.

a Participants with total bilirubin levels of 0.5–0.7 mg/dl were used as the referent group for comparison. All data were adjusted for survey period, age, sex, race/ethnicity, body mass index, education, smoking, regular alcohol consumption, history of cardiovascular disease, diabetes, albuminuria, cancer, fibrates, angiotensin-converting enzyme inhibitors/angiotensin receptor blockers, diuretics, calcium channel blockers, high-density lipoprotein cholesterol, serum albumin, blood urea nitrogen, estimated glomerular filtration rate, C-reactive protein, alkaline phosphatase, alanine aminotransferase, aspartate aminotransferase, γ-glutamyltransferase, uric acid, white blood cell count, and hemoglobin.

## Discussion

This study investigated the relationship between total bilirubin levels and total mortality after a mean follow-up period of 4.5 years in a national representative population of older men and women. The results show that the association of total bilirubin levels with total mortality was non-linear, which is consistent with a previous report by Horsfall et al [12]. In both studies, low total bilirubin levels were significantly associated with a higher mortality risk. Of note, there was also a tendency for a total bilirubin level of ≥0.8 mg/dl to be associated with a higher mortality risk, compared to a level of 0.5–0.7 mg/dl in the present study, especially in non-Hispanic Whites and never smokers.

The association of lower total bilirubin levels with higher total mortality risk is expected as bilirubin has anti-oxidant and anti-inflammatory effects [1]. This is consistent with other previous studies [10]–[12]. Genetic variants associated with higher total bilirubin levels have been shown to be associated with lower risk of total mortality, suggesting a causal relationship between total bilirubin and mortality risk [22]. In the recent study of Horsfall et al, lower total bilirubin levels were associated with a higher risk of any CVD event, coronary heart disease, myocardial infarction, and all-cause mortality [12]. An inverse relationship between total bilirubin and CVD mortality has also been reported [23]. However, in our present study, we did not observe any significant association of lower total bilirubin levels with higher risk of CVD mortality. In this regard, conflicting results on the relationship between total bilirubin and CVD have been reported. A recent study of 43,708 people in Denmark demonstrated that the inverse association of total bilirubin with ischemic heart disease and myocardial infarction becomes not significant after further adjustment for more cardiovascular risk factors [24]. Meta-analysis of studies involving 14,711 case and 60,324 controls also has not revealed a causal relationship between total bilirubin and cardiovascular diseases [24]. Another recent study has shown that genetic variants, associated with total bilirubin levels, are associated with total mortality, but not vascular calcified plaque and CVD mortality [25]. The non-significant association between total bilirubin and CVD mortality in that study and our present study could be due to the low number of CVD deaths and thus insufficient power. Nevertheless, our study suggests that lower levels of total bilirubin may be more related to non-CVD mortality, such as cancer mortality, than CVD mortality.

It is unclear why higher total bilirubin levels were associated with higher mortality risk, especially in in non-Hispanic Whites and never smokers in this study. It is interesting that some previous studies have demonstrated an association of higher total bilirubin levels with higher CVD and total mortality risk. For example, a non-linear U-shaped relationship between total bilirubin levels and risk of incident CAD has been reported in a previous study of 216 CAD patients and 434 matched controls with a 5-year follow-up [26]. In the British Regional Heart Study, a prospectively study of 7,685 middle-aged British men with a 11.5 year of follow-up, a U-shaped relationship between total bilirubin and ischemic heart disease was also found [21]. In the Simvastatin and Ezetimibe in Aortic Stenosis study, a randomized trial studying the effects of simvastatin and ezetimibe therapy on cardiovascular adverse events in 1,873 patients with mild-to-moderate, asymptomatic aortic stenosis, higher total bilirubin levels were associated with higher total mortality risk [27]. In another study of patients with ST-elevation myocardial infarction (STEMI), high total bilirubin levels were associated with higher risk of cardiovascular mortality, advanced heart failure, and adverse cardiac events after primary coronary intervention [28]. In fact, total bilirubin levels have been reported to be elevated, probably due to an increase in stress-induced HO-1 activity after acute myocardial infarction [29], and total bilirubin levels are positively associated with the severity of coronary artery disease in patients with both STEMI [30], and non-STEMI [31]. Therefore, the association of high total bilirubin levels with higher total mortality risk could be a reflection of stress-induced activation of HO-1 increasing total bilirubin levels as a protective mechanism.

Total bilirubin levels are often elevated in liver diseases and may therefore contribute to the association with higher mortality risk. However, it should be noted that participants with abnormal liver function were excluded from the present study and that there were only 3 deaths from liver disease (including viral hepatitis) among the 692 participants with identified causes of death.

Another possible explanation for the association of higher total bilirubin levels with higher mortality risk could be due the confounding effect by indication, with the participants that had been diagnosed with CVD being at increased risk of mortality from CVD. That is, as participants with self-reported CVD are likely to be taking lipid-lowering medications, especially statins, to reduce cardiovascular events, they tended to have higher total bilirubin levels as a consequence of statin-mediated stimulation of HO-1 activity [32]–[34]. This hypothesis was supported, at least in part, by the finding that the prevalence of self-reported history of CVD was the highest in patients with the highest total bilirubin levels. There was also a non-significant trend of higher usage of lipid-lowering medication (mainly statins) with high bilirubin levels (Supplementary Table S1). This confounding effect by indication may also explain the stronger relationship of higher total bilirubin levels with higher mortality risk in non-Hispanic Whites, who had the highest prevalence of self-reported CVD and usage of lipid-lowering medications (Supplementary Table S4). Notably, higher levels of total bilirubin also tended to be associated with higher risk of mortality not related to CVD and cancer. Further studies with larger sample sizes are needed to elucidate the association of higher total bilirubin levels with different cause-specific mortality risk.

In a sub-group analysis, a more linear association of lower total bilirubin levels with higher total mortality risk was found in current smokers. A previous study of NHANES data has demonstrated a stronger inverse association between total bilirubin and peripheral arterial disease in current smokers than non-smokers [7]. As smoking can increase inflammation and oxidative stress, it is possible that bilirubin may offer more protection in smokers, than non-smokers. In fact, total bilirubin levels are lower in smokers and decrease with increasing smoking amount and duration [9], [35]. A recent study found a significant, positive association of total bilirubin levels with lung function in smokers with high BMI, but not in non-smokers [36].

The major limitations of this study include the small sample size, the low number of deaths and the short follow-up period, all of which limit the study power. This also makes the analysis of the relationship of total bilirubin levels with cause-specific mortality, other than CVD and cancer mortality, not feasible. Common genetic variants, such as those in the gene encoding the hepatic enzyme uridine diphosphate-glucuronosyltransferase 1 (*UGT1A1*), have been shown to affect total bilirubin levels, and thus may confound the results [1], [22], [24], [25]. Despite these limitations, our study has the advantage of using data from NHANES with an appropriate sampling design, quality control, and nationally representative estimates. The availability of data on different covariates helps to minimize their confounding effect by adjustment in multivariable regression model.

In conclusion, in this study of a national representative sample of older adults from the general population, a non-linear association was found between total bilirubin levels and total mortality. Further studies are needed to elucidate potential mechanism of this association.

## Supporting Information

Table S1 **Clinical Characteristics in United States Older Adults by Total Bilirubin Levels, 1999-2004.**
(DOCX)

Table S2 **Association of Total Bilirubin Levels With Mortality From CVD, Cancer, and Other Causes in United States Older Adults, 1999–2004.**
(DOCX)

Table S3 **Subgroup Analysis for the Associations of Lower and Higher Total Bilirubin Levels with Total Mortality in United States Older Adults, 1999–2004.**
(DOCX)

Table S4 **Self-reported CVD and Lipid-lowering Medication Use by Race/ethnicity in United States Older Adults, 1999–2004.**
(DOCX)
