# Supplementary material for: The Relationship between Total Bilirubin Levels and Total Mortality in Older Adults: The United States National Health and Nutrition Examination Survey (NHANES) 1999-2004
Source: PLoS One. 2014 Apr 11;9(4):e94479. doi: 10.1371/journal.pone.0094479 (PMC3984185; doi:10.1371/journal.pone.0094479)
Supplement: Table S1 — Clinical Characteristics in United States Older Adults by Total Bilirubin Levels, 1999-2004. (DOCX) [file pone.0094479.s001.docx]

**Supplementary Table S1.** Clinical Characteristics in United States Older Adults by Total Bilirubin Levels, 1999-2004.

| **Characteristics** | **n** | **Total bilirubin, mg/dl** | | | | | | **P for trend^c^** |
| --- | --- | --- | --- | --- | --- | --- | --- | --- |
|  |  | **0.1-0.4**  **(n=605)** | **0.5**  **(n=655)** | **0.6**  **(n=952)** | **0.7**  **(n=669)** | **0.8-0.9**  **(n=832)** | **≥1.0**  **(n=590)** |  |
| Demographic and lifestyle factors |  |  |  |  |  |  |  |  |
| Age, y | 4,303 | 70.2 (0.4) | 70.7 (0.3) | 71.3 (0.3) | 71.4 (0.5) | 71.6 (0.4) | 71.3 (0.3) | 0.002 |
| Women, % | 4,303 | 74.8 (1.7) | 65.4 (2.2) | 66.0 (1.8) | 54.5 (2.5) | 45.7 (2.1) | 32.9 (2.8) | <0.001 |
| BMI, kg/m^2^ | 4,097 | 28.3 (0.3) | 28.1 (0.3) | 28.6 (0.2) | 28.3 (0.3) | 28.2 (0.2) | 27.5 (0.3) | 0.36 |
| Race/ethnicity, % |  |  |  |  |  |  |  | 0.015 |
| Non-Hispanic White | 2,511 | 77.0 (3.6) | 79.0 (2.4) | 78.7 (2.5) | 84.3 (1.9) | 86.6 (2.0) | 85.4 (2.7) |  |
| Non-Hispanic Black | 655 | 12.8 (2.3) | 9.5 (1.4) | 8.8 (1.5) | 7.2 (1.2) | 4.6 (1.0) | 3.9 (0.7) |  |
| Mexican American | 879 | 2.9 (0.8) | 3.9 (0.9) | 2.9 (0.7) | 3.0 (0.9)^d^ | 3.2 (0.9) | 2.8 (0.8) |  |
| Others | 258 | 7.3 (3.6)^d^ | 7.6 (1.6) | 9.5 (1.8) | 5.5 (1.2) | 5.6 (1.4) | 7.9 (2.1) |  |
| Education, % |  |  |  |  |  |  |  | 0.051 |
| <High school | 1,802 | 34.1 (2.2) | 34.4 (2.9) | 29.6 (1.9) | 29.4 (3.0) | 27.1 (2.3) | 25.5 (1.9) |  |
| High school diploma | 1,009 | 28.7 (2.9) | 31.7 (2.7) | 31.3 (2.2) | 28.6 (2.3) | 26.5 (1.7) | 26.5 (2.7) |  |
| >High school | 1,476 | 37.2 (2.8) | 33.9 (3.2) | 39.1 (2.6) | 42.1 (2.6) | 46.4 (2.2) | 48.0 (2.8) |  |
| Smoking, % |  |  |  |  |  |  |  | <0.001 |
| Never | 2,024 | 48.6 (3.2) | 46.0 (1.9) | 49.8 (2.5) | 46.0 (2.7) | 46.8 (2.3) | 42.7 (2.5) |  |
| Former | 1,765 | 37.0 (3.6) | 38.4 (2.4) | 35.7 (2.0) | 42.4 (2.6) | 46.3 (2.2) | 51.4 (2.2) |  |
| Current | 505 | 14.4 (1.9) | 15.6 (1.8) | 14.5 (1.9) | 11.6 (1.4) | 6.9 (1.0) | 5.9 (1.0) |  |
| Regular alcohol consumption, % | 4,106 | 20.0 (2.2) | 21.7 (2.8) | 20.6 (2.0) | 25.4 (2.9) | 30.4 (2.1) | 36.3 (2.4) | <0.001 |
|  |  |  |  |  |  |  |  |  |
| Chronic conditions |  |  |  |  |  |  |  |  |
| History of CVD, % | 4,186 | 22.7 (2.3) | 25.7 (2.2) | 24.8 (2.0) | 23.4 (1.9) | 21.9 (1.8) | 25.9 (2.5) | 0.020 |
| Diabetes, % | 4,298 | 18.8 (3.1) | 22.5 (1.9) | 19.9 (1.5) | 18.1 (1.8) | 15.6 (1.8) | 13.1 (1.7) | 0.001 |
| Hypertension, % | 4,110 | 68.6 (2.3) | 68.2 (2.3) | 70.0 (2.1) | 62.8 (3.0) | 62.5 (2.2) | 66.0 (3.1) | 0.32 |
| Albuminuria,% | 4,169 | 23.6 (2.4) | 20.7 (2.8) | 19.5 (1.5) | 17.5 (1.8) | 19.5 (1.7) | 21.1 (2.2) | 0.51 |
| Arthritis, % | 4,292 | 55.8 (3.1) | 51.7 (1.9) | 49.6 (2.4) | 47.6 (2.8) | 49.4 (2.2) | 44.5 (2.7) | 0.23 |
| Cancer, % | 4,298 | 18.8 (2.1) | 17.9 (2.0) | 20.3 (1.5) | 19.4 (2.0) | 25.1 (1.6) | 24.2 (2.2) | 0.59 |
| Lipid-lowering medication, % | 4,296 | 22.8 (2.2) | 24.6 (2.1) | 26.2 (1.6) | 26.9 (2.2) | 27.0 (2.2) | 30.9 (2.3) | 0.40 |
| Statin, % | 4,296 | 21.0 (2.1) | 22.6 (2.1) | 23.2 (1.5) | 25.6 (2.4) | 25.2 (2.3) | 28.1 (2.0) | 0.66 |
| Fibrate, % | 4,296 | 1.3 (0.5)^d^ | 0.9 (0.4)^d^ | 3.2 (0.9) | 1.4 (0.4) | 1.6 (0.5) | 1.8 (0.9)^d^ | 0.35 |
| Nicotinic acid, % | 4,296 | 0.0 (0.0)^d^ | 0.2 (0.2)^d^ | 0.8 (0.3)^d^ | 0.6 (0.4)^d^ | 0.5 (0.3)^d^ | 0.5 (0.3)^d^ | 0.40 |
| Others, %^a^ | 4,296 | 0.7 (0.4)^d^ | 1.1 (0.4)^d^ | 1.5 (0.5)^d^ | 0.8 (0.4)^d^ | 1.1 (0.5)^d^ | 1.6 (0.8)^d^ | 0.71 |
| Anti-hypertensive medication, % | 4,296 | 54.8 (2.7) | 54.3 (2.1) | 58.0 (2.7) | 57.1 (2.8) | 55.3 (2.0) | 58.9 (3.1) | 0.64 |
| ACEI/ARB | 4,296 | 26.7 (1.6) | 26.0 (2.1) | 28.2 (2.4) | 28.4 (2.1) | 28.8 (1.7) | 32.2 (2.8) | 0.40 |
| Diuretic | 4,296 | 23.8 (2.5) | 24.5 (2.0) | 27.7 (1.6) | 28.1 (2.5) | 24.8 (1.3) | 25.5 (2.6) | 0.40 |
| β-blocker | 4,296 | 15.5 (2.5) | 17.5 (1.8) | 18.2 (2.0) | 18.3 (1.9) | 20.7 (1.7) | 22.9 (2.1) | 0.49 |
| CCB | 4,296 | 22.0 (2.1) | 20.1 (2.0) | 20.0 (1.8) | 19.2 (1.7) | 16.8 (1.8) | 13.0 (1.4) | 0.003 |
| Others | 4,296 | 12.1 (2.1) | 8.8 (1.1) | 9.9 (1.3) | 10.1 (1.3) | 12.0 (1.4) | 13.0 (2.0) | 0.33 |
|  |  |  |  |  |  |  |  |  |
| Clinical biocmarkers |  |  |  |  |  |  |  |  |
| Total cholesterol, mg/dl | 4,297 | 217.9 (1.8) | 216.1 (2.1) | 211.5 (1.8) | 211.0 (1.8) | 207.7 (1.3) | 201.8 (2.1) | 0.41 |
| HDL cholesterol, mg/dl | 4,294 | 55.9 (1.1) | 53.8 (0.8) | 54.5 (0.6) | 54.7 (0.7) | 53.4 (0.8) | 53.3 (0.8) | 0.040 |
| Serum albumin, g/dl | 4,303 | 4.22 (0.02) | 4.22 (0.02) | 4.18 (0.01) | 4.24 (0.02) | 4.24 (0.01) | 4.30 (0.01) | <0.001 |
| Blood urea nitrogen, mg/dl^b^ | 4,303 | 15.9 (15.4-16.4) | 15.9 (15.3-16.5) | 15.9 (15.3-16.5) | 15.4 (14.8-16.1) | 15.5 (15.1-16.0) | 15.6 (15.1-16.1) | 0.063 |
| eGFR, ml/min/1.73m^2^ | 4,303 | 72.0 (1.1) | 69.4 (0.9) | 69.8 (0.7) | 69.2 (0.8) | 70.2 (0.9) | 69.4 (0.8) | 0.90 |
| C-reactive protein, mg/dl^b^ | 4,301 | 0.38 (0.34-0.42) | 0.31 (0.28-0.34) | 0.28 (0.26-0.30) | 0.26 (0.24-0.29) | 0.21 (0.19-0.23) | 0.19 (0.17-0.22) | <0.001 |
| Alkaline phosphatase, U/l^b^ | 4,303 | 77.5 (74.6-80.6) | 74.7 (72.4-77.0) | 71.8 (69.8-73.9) | 70.3 (68.2-72.5) | 68.5 (66.6-70.5) | 68.1 (66.7-69.5) | 0.010 |
| Alanine aminotransferase, U/l^b^ | 4,303 | 18.4 (17.7-19.2) | 19.1 (18.3-19.8) | 19.5 (18.9-20.1) | 20.7 (20.0-21.4) | 20.7 (20.3-21.2) | 21.3 (20.5-22.1) | <0.001 |
| Aspartate aminotransferase, U/l^b^ | 4,303 | 21.4 (20.7-22.2) | 21.9 (21.4-22.3) | 22.6 (22.2-23.0) | 23.4 (22.8-24.1) | 23.5(23.0-24.1) | 24.3 (23.7-25.0) | <0.001 |
| γ-glutamyltransferase, U/l^b^ | 4,303 | 20.8 (19.4-22.2) | 21.5 (20.2-22.9) | 21.2 (20.3-22.1) | 22.0 (21.1-22.9) | 21.0 (20.0-22.2) | 23.1 (22.1-24.2) | 0.065 |
| Uric acid, mg/dl | 4,303 | 5.30 (0.10) | 5.53 (0.07) | 5.62 (0.07) | 5.70 (0.08) | 5.76 (0.06) | 6.04 (0.07) | 0.002 |
| White blood cell count, 10^3^cells/µl^b^ | 4,300 | 7.14 (6.87-7.44) | 6.96 (6.75-7.18) | 6.95 (6.79-7.11) | 6.94 (6.73-7.16) | 6.63 (6.52-6.75) | 6.52 (6.32-6.72) | <0.001 |
| Hemoglobin, g/dl | 4,300 | 13.5 (0.1) | 13.9 (0.1) | 13.9 (0.1) | 14.4 (0.1) | 14.6 (0.1) | 14.9 (0.1) | <0.001 |

ACEI = angiotensin-converting enzyme inhibitor; ARB = angiotensin receptor blocker; BMI = body mass index; CCB = calcium channel blocker; CVD = cardiovascular disease; eGFR = estimated glomerular filtration rate; HDL = high-density lipoprotein.

Data are expressed as mean or percent (standard error), unless otherwise noted.

^a^Includes bile acid sequestrants, cholesterol adsorption inhibitors, and other types of lipid-lowering medications.

^b^Data are expressed as geometric mean (95% confidence interval) and were log-transformed before analysis.

^c^Estimated from multivariable linear, logistic or ordinal regression after adjusting for age, sex, race/ethnicity, and survey period, where appropriate, in which categorical levels of total bilirubin were entered as a continuous independent variable in the model.

^d^Estimates are unreliable due to coefficient of variation > 0.3.
