# Supplementary material for: The Relationship between Total Bilirubin Levels and Total Mortality in Older Adults: The United States National Health and Nutrition Examination Survey (NHANES) 1999-2004
Source: PLoS One. 2014 Apr 11;9(4):e94479. doi: 10.1371/journal.pone.0094479 (PMC3984185; doi:10.1371/journal.pone.0094479)
Supplement: Table S2 — Association of Total Bilirubin Levels With Mortality From CVD, Cancer, and Other Causes in United States Older Adults, 1999–2004. (DOCX) [file pone.0094479.s002.docx]

**Supplementary Table S2.** Association of Total Bilirubin Levels With Mortality From CVD, Cancer, and Other Causes in United States Older Adults, 1999-2004.

| **Total bilirubin, mg/dl** | **Model 1^a^** | | **Model 2^b^** | | **Model 3^c^** | | **Model 4^d^** | |
| --- | --- | --- | --- | --- | --- | --- | --- | --- |
|  | **HR (95% CI)** | **P** | **HR (95% CI)** | **P** | **HR (95% CI)** | **P** | **HR (95% CI)** | **P** |
| CVD death |  |  |  |  |  |  |  |  |
| 0.1-0.4 | 1.32 (0.80-2.16) | 0.27 | 1.29 (0.74-2.26) | 0.36 | 1.30 (0.81-2.08) | 0.26 | 1.17 (0.69-1.99) | 0.54 |
| 0.5-0.7 | 1.00 (referent) |  | 1.00 (referent) |  | 1.00 (referent) |  | 1.00 (referent) |  |
| ≥0.8 | 0.78 (0.51-1.21) | 0.26 | 0.92 (0.57-1.48) | 0.72 | 0.86 (0.55-1.37) | 0.52 | 0.92 (0.58-1.47) | 0.72 |
| Overall P |  | 0.34 |  | 0.63 |  | 0.49 |  | 0.80 |
|  |  |  |  |  |  |  |  |  |
| Cancer death |  |  |  |  |  |  |  |  |
| 0.1-0.4 | 2.00 (1.24-3.24) | 0.006 | 2.04 (1.22-3.40) | 0.007 | 2.23 (1.33-3.73) | 0.003 | 1.94 (1.14-3.31) | 0.0216 |
| 0.5-0.7 | 1.00 (referent) |  | 1.00 (referent) |  | 1.00 (referent) |  | 1.00 (referent) |  |
| ≥0.8 | 0.92 (0.59-1.43) | 0.69 | 0.99 (0.61-1.60) | 0.95 | 0.84 (0.50-1.43) | 0.52 | 0.99 (0.57-1.71) | 0.96 |
| Overall P |  | 0.0162 |  | 0.0263 |  | 0.010 |  | 0.056 |
|  |  |  |  |  |  |  |  |  |
| Others |  |  |  |  |  |  |  |  |
| 0.1-0.4 | 1.17 (0.81-1.71) | 0.39 | 1.26 (0.82-1.92) | 0.28 | 1.40 (0.93-2.11) | 0.10 | 1.23 (0.82-1.85) | 0.32 |
| 0.5-0.7 | 1.00 (referent) |  | 1.00 (referent) |  | 1.00 (referent) |  | 1.00 (referent) |  |
| ≥0.8 | 1.24 (0.92-1.67) | 0.16 | 1.50 (1.08-2.08) | 0.0172 | 1.63 (1.14-2.32) | 0.008 | 1.88 (1.27-2.76) | 0.002 |
| Overall P |  | 0.34 |  | 0.060 |  | 0.0283 |  | 0.009 |

CI = confidence interval; CVD = cardiovascular disease; HR = hazard ratio.

^a^Adjusted for survey period, age, sex, and race/ethnicity (n=4,295).

^b^Further adjusted for body mass index, education, smoking, and regular alcohol consumption (n=3,922).

^c^Further adjusted for history of CVD, diabetes, albuminuria, cancer, fibrates, angiotensin-converting enzyme inhibitors/angiotensin receptor blockers, diuretics, and calcium channel blockers (n=3,758).

^d^Further adjusted for HDL cholesterol, serum albumin, blood urea nitrogen, estimated glomerular filtration rate, C-reactive protein, alkaline phosphatase, alanine aminotransferase, aspartate aminotransferase, γ-glutamyltransferase, uric acid, white blood cell count, and hemoglobin (n=3,752).
