# Supplementary material for: The Relationship between Total Bilirubin Levels and Total Mortality in Older Adults: The United States National Health and Nutrition Examination Survey (NHANES) 1999-2004
Source: PLoS One. 2014 Apr 11;9(4):e94479. doi: 10.1371/journal.pone.0094479 (PMC3984185; doi:10.1371/journal.pone.0094479)
Supplement: Table S3 — Subgroup Analysis for the Associations of Lower and Higher Total Bilirubin Levels with Total Mortality in United States Older Adults, 1999–2004. (DOCX) [file pone.0094479.s003.docx]

**Supplementary Table S3.** Subgroup Analysis for the Associations of Lower and Higher Total Bilirubin Levels with Total Mortality in United States Older Adults, 1999-2004.^a^

| **Subgroup** | **n** | **Mortality rate, % (SE)** | **0.1-0.4 mg/dl** | | **≥0.8 mg/dl** | | **Overall P** | **P for interaction^b^** |
| --- | --- | --- | --- | --- | --- | --- | --- | --- |
|  |  |  | **HR (95% CI)** | **P** | **HR (95% CI)** | **P** |  |  |
| Age, year |  |  |  |  |  |  |  |  |
| 60-70 | 2055 | 6.0 (0.7) | 1.21 (0.61-2.38) | 0.58 | 1.04 (0.57-1.89) | 0.89 | 0.86 | 0.94 |
| ≥71 | 2248 | 22.7 (1.0) | 1.31 (1.01-1.70) | 0.043 | 1.31 (0.98-1.75) | 0.071 | 0.017 |  |
| Gender |  |  |  |  |  |  |  |  |
| Men | 2101 | 17.1 (1.2) | 1.15 (0.77-1.71) | 0.48 | 0.98 (0.71-1.33) | 0.88 | 0.74 | 0.29 |
| Women | 2202 | 12.0 (0.7) | 1.54 (1.10-2.15) | 0.012 | 1.68 (1.16-2.43) | 0.008 | 0.003 |  |
| Regular alcohol consumption |  |  |  |  |  |  |  |  |
| No | 3157 | 15.1 (0.9) | 1.37 (1.06-1.77) | 0.016 | 1.30 (1.03-1.65) | 0.027 | 0.005 | 0.61 |
| Yes | 949 | 10.6 (1.0) | 1.07 (0.50-2.25) | 0.87 | 0.99 (0.51-1.92) | 0.97 | 0.98 |  |

CI = confidence interval; HR = hazard ratio.

^a^Participants with total bilirubin levels of 0.5-0.7 mg/dl were used as the referent group for comparison. All data were adjusted for survey period, age, sex, race/ethnicity, body mass index, education, smoking, regular alcohol consumption, history of cardiovascular disease, diabetes, albuminuria, cancer, fibrates, angiotensin-converting enzyme inhibitors/angiotensin receptor blockers, diuretics, calcium channel blockers, high-density lipoprotein cholesterol, serum albumin, blood urea nitrogen, estimated glomerular filtration rate, C-reactive protein, alkaline phosphatase, alanine aminotransferase, aspartate aminotransferase, γ-glutamyltransferase, uric acid, white blood cell count, and hemoglobin.

^b^For the subgroups by age, the categorical subgroup variable, instead of the continuous level, was used in the adjustment model when assessing the P value for interaction.
