# Supplementary material for: The Relationship between Total Bilirubin Levels and Total Mortality in Older Adults: The United States National Health and Nutrition Examination Survey (NHANES) 1999-2004
Source: PLoS One. 2014 Apr 11;9(4):e94479. doi: 10.1371/journal.pone.0094479 (PMC3984185; doi:10.1371/journal.pone.0094479)
Supplement: Table S4 — Self-reported CVD and Lipid-lowering Medication Use by Race/ethnicity in United States Older Adults, 1999–2004. (DOCX) [file pone.0094479.s004.docx]

**Supplementary Table S4.** Self-reported CVD and Lipid-lowering Medication Use by Race/ethnicity in United States Older Adults, 1999-2004.

| **Subgroup** | **n** | **CVD** | | **Any lipid-lowering medication** | | **Statins** | |
| --- | --- | --- | --- | --- | --- | --- | --- |
|  |  | **% (SE)** | **OR (95% CI)** | **% (SE)** | **OR (95% CI)** | **% (SE)** | **OR (95% CI)** |
| Non-Hispanic White | 2511 | 24.7 (1.1) | 1.00 (referent) | 28.0 (1.0) | 1.00 (referent) | 25.7 (1.0) | 1.00 (referent) |
| Non-Hispanic Black | 655 | 24.2 (2.1) | 1.06 (0.81-1.38) | 17.9 (1.9) | 0.56 (0.42-0.75) | 17.2 (1.8) | 0.60 (0.45-0.80) |
| Mexican American | 879 | 18.8 (1.4) | 0.79 (0.64-0.98) | 17.9 (1.5) | 0.55 (0.45-0.66) | 16.4 (1.2) | 0.56 (0.47-0.66) |
| Others | 258 | 18.4 (3.3) | 0.79 (0.51-1.21) | 20.5 (3.6) | 0.68 (0.43-1.09) | 18.6 (3.0) | 0.68 (0.45-1.02) |
| Overall P |  |  | 0.11 |  | <0.001 |  | <0.001 |

CI = confidence interval; CVD = cardiovascular disease; OR = odds ratio; SE = standard error.

*P* values were estimated from multivariable logistic regression after adjusting for age, sex, race/ethnicity, and survey period, where appropriate.
